# Confocal reference free traction force microscopy

**DOI:** 10.1038/ncomms12814

**Published:** 2016-09-29

**Authors:** Martin Bergert, Tobias Lendenmann, Manuel Zündel, Alexander E. Ehret, Daniele Panozzo, Patrizia Richner, David K. Kim, Stephan J. P. Kress, David J. Norris, Olga Sorkine-Hornung, Edoardo Mazza, Dimos Poulikakos, Aldo Ferrari

**Affiliations:** 1 ETH Zurich, Laboratory of Thermodynamics in Emerging Technologies, Sonneggstrasse 3, 8092 Zurich, Switzerland; 2 ETH Zurich, Institute for Mechanical Systems, Leonhardstrasse 21, 8092 Zurich, Switzerland; 3 Empa, Swiss Federal Laboratories for Materials Science and Technology, Überlandstrasse 129, 8600 Dübendorf, Switzerland; 4 ETH Zurich, Institute for Visual Computing, Interactive Geometry Lab, Universitätstrasse 6, 8092 Zurich, Switzerland; 5 Courant Institute of Mathematical Sciences, New York University, 719 Broadway, New York 10003, USA; 6 ETH Zurich, Optical Materials Engineering Laboratory, Leonhardstrasse 21, 8092 Zurich, Switzerland

## Abstract

The mechanical wiring between cells and their surroundings is fundamental to the regulation of complex biological processes during tissue development, repair or pathology. Traction force microscopy (TFM) enables determination of the actuating forces. Despite progress, important limitations with intrusion effects in low resolution 2D pillar-based methods or disruptive intermediate steps of cell removal and substrate relaxation in high-resolution continuum TFM methods need to be overcome. Here we introduce a novel method allowing a one-shot (live) acquisition of continuous in- and out-of-plane traction fields with high sensitivity. The method is based on electrohydrodynamic nanodrip-printing of quantum dots into confocal monocrystalline arrays, rendering individually identifiable point light sources on compliant substrates. We demonstrate the undisrupted reference-free acquisition and quantification of high-resolution continuous force fields, and the simultaneous capability of this method to correlatively overlap traction forces with spatial localization of proteins revealed using immunofluorescence methods.

## Abstract (web summary)

Traction force microscopy is an effective method of measuring forces between cells and their environment, but requires removing the cells to obtain a reference image. Here the authors use nanodrip printing of quantum dots into compliant substrates to provide a regular array of fiducial spots, removing the need for a reference image.

## Graphical abstract

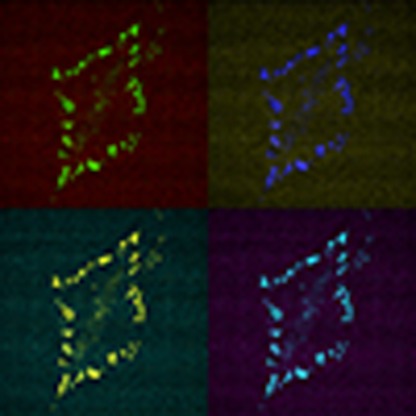

Cells exert forces on their environment, mainly but not exclusively through the contractile acto-myosin machinery. These forces are transmitted to the extracellular surroundings through integrin-based adhesions (that is, focal adhesions) and enable shape changes and directional migration^1^. The biological function of this mechanical machinery goes beyond the simple physical anchoring and conveys environmental signals, which cells sense and respond to^2^.

Traction force microscopy (TFM) provides a powerful tool to experimentally access cellular forces. In the last two decades multiple protocols for the measurement of cell-generated tractions have been developed, based on the optical detection of force-dependent deformations of compliant substrates^3^,^4^,^5^. Of these, lower-resolution discrete methods such as arrays of elastic micro-posts^6^,^7^ allow the estimation of forces from a single image, yet carry significant limitations to the detection of out-of-plane components of traction forces, as well as artefacts due to their non-continuous, structured and intrusive nature^5^,^8^,^9^,^10^. Continuum TFM methods exploit elastic substrates containing randomly dispersed fluorescent beads^11^,^12^,^13^. These approaches yield high-resolution in- and out-of-plane force maps but require the additional acquisition of a reference (load-free) image, typically captured on cell removal and destruction, which markedly complicates experimental procedures and precludes post-processing such as the colocalization with immunofluorescence staining. Attempts to bypass these shortcomings, by applying micro-patterning of adhesive islands^14^,^15^ or lithographic photoresist into ordered arrays^16^, are hampered by major drawbacks such as the poor spatial resolution or the introduction of intrusive topographical features. Molecular methods, such as DNA-based force sensors^17^, entail very high spatial resolution (about 200 nm) and one-shot force magnitude detection on flat, non-intrusive surfaces, but are unable to discern any force directionality.

Here we use highly precise electrohydrodynamic nanodrip-printing of quantum dots (QDs)^18^,^19^,^20^,^21^ into monocrystalline, confocal arrays on elastomeric substrates and introduce a high-resolution and reference-free method (called confocal TFM or cTFM), capable of in- and out-of-plane force detection, which takes advantage of many assets of previously developed approaches while significantly advancing the landscape of reference-free force detection in cell biology and medicine.

## Results

### Design of the cTFM platform

**Figure 1:**
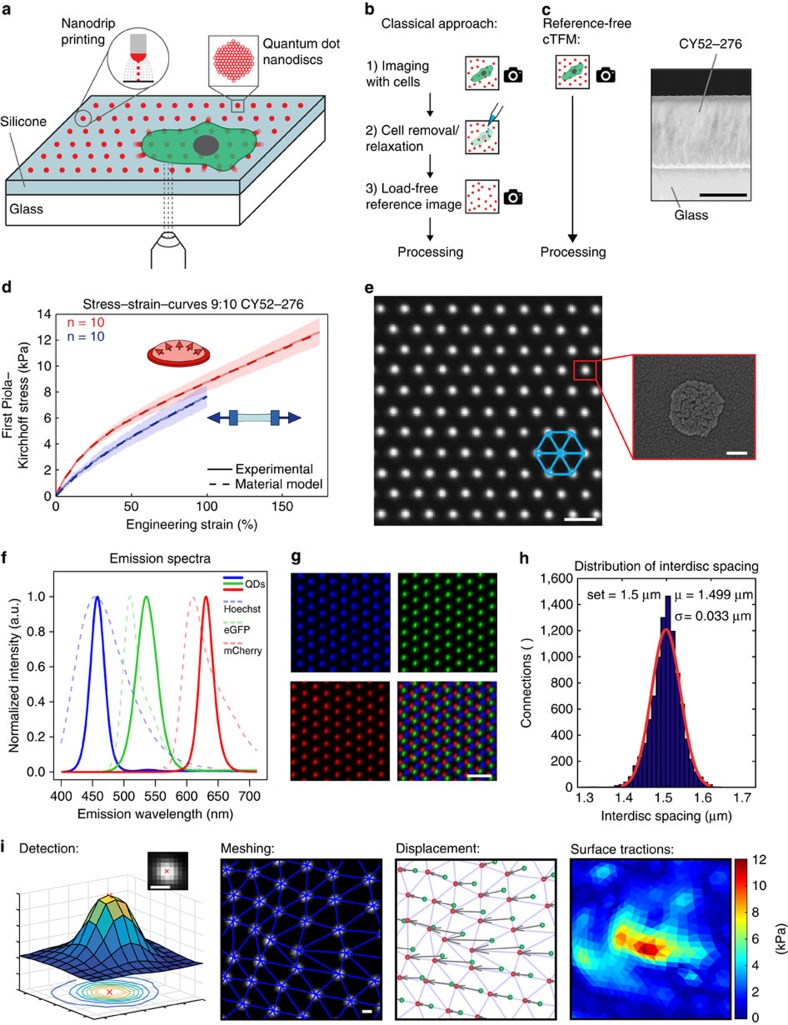
Overview and characterization of the cTFM platform. (**a**) Schematic of the cTFM set-up and involved techniques. (**b**) Workflow of the classical continuum approach versus the one-step cTFM process. (**c**) Scanning electron microscopy (SEM) image of cross-section of elastic silicone layer on glass coverslip. Scale bar, 20 μm. (**d**) Results of tensile (uniaxial) and inflation (equibiaxial) testing of CY52-276 silicone mixed at a 9:10 ratio. Free sample dimensions 40 × 10 mm (uniaxial) and diameter 30 mm (inflation). Thicknesses were in the range from 0.5 to 0.75 mm; exact thickness was measured for each sample independently (see Methods). Shaded area: s.d.; *n*=10 samples per test. (**e**) Representative example (*n*=20) of QD nanodisc printing on elastic silicone substrates. Scale bar, 2 μm. Red box: SEM image of QD nanodisc on glass. Scale bar, 100 nm. (**f**) Emission spectra of blue, green and red QDs, as well as Hoechst, eGFP and mCherry for comparison. (**g**) Blue, green and red QDs were printed on the same substrate. Lower right image shows the merging of the three colours. Scale bar, 3 μm. (**h**) Representative distribution of actual spacing between fluorescent QD nanodiscs after printing. (**i**) Overview of the analysis procedure. Subpixel detection of the QD nanodisc centre (Detection), followed by computational reconstruction of the triangular mesh (Meshing). From the displacement field (Displacement) the surface tractions are reconstructed using FEA (Surface tractions). Scale bars, 500 nm.

The cTFM set-up in Fig. 1a–c is comprised of a typical, 170 μm glass coverslip spin-coated with a ≈30 μm thick layer of a highly deformable CY52-276 silicone (Dow Corning)^22^,^23^,^24^,^25^. The CY52-276 silicone is a two-component elastomer yielding bulk elastic moduli ranging from 1 to 20 kPa for mixing ratios close to 1:1 (Supplementary Fig. 1a). Standard PDMS (Sylgard 184) requires mixing ratios of around 1:60 to yield stiffnesses in the same range^26^,^27^. Silicones in general are very viscous and sticky before curing, and handling small amounts proves to be difficult. Thus, substrate preparation, especially for the small volumes required for spin-coating applications (ml regime), is simpler and more reliable with CY52-276 than for Sylgard 184. CY52-276 has a refractive index of *n*_632nm_=1.403 (ref. 28) and is optically transparent (Supplementary Fig. 1b). The point spread function measured in standard fluorescence wide-field microscopy is similar to the one obtained with commonly used polyacrylamide gels (Supplementary Fig. 1c–e). A unit of 0.05% poly(dimethylsiloxane-*b*-ethylene oxide) is added during silicone fabrication to increase hydrophilicity^29^, which aids nanodrip-printing precision^18^,^19^. All cTFM analyses shown in this work use a CY52-276 mixing ratio of 9:10, which provides an elastic modulus of ∼12.6 kPa according to our mechanical tests. A nonlinear hyperelastic material model, required for the analysis of large deformations of the substrate, is defined based on a comprehensive mechanical characterization of thin-cast samples in multi-axial mechanical tests^30^ (Fig. 1d and Supplementary Fig. 1f; for details see Methods). In addition, cyclic uniaxial strain rate sweep tests were performed between 0.3% and 5.2% s^−1^ at large strains, and showed negligible rate dependence of the material response (Supplementary Fig. 1g).

The silicone surface features a monocrystalline array of fluorescent nanodiscs, so that the lines connecting nearest neighbours form a grid of equilateral triangles (Fig. 1e). Each printed fluorescent nanodisc contains a countable number of custom-made QDs^20^ (Supplementary Movie 1). Individual nanodiscs are about 200 nm in diameter (Fig. 1e) and ≤30 nm in thickness and thus represent confocal point sources of light, emitting a bright and stable fluorescent signal (Supplementary Fig. 2), without introducing intrusive surface topography^31^. By varying the size and composition of the QDs (see Methods for details), fluorescent nanodiscs with various and well-defined emission spectra can be created (Fig. 1f), comparable and compatible with standard live-cell fluorescent proteins (for example, green fluorescent protein or mCherry). Sequential nanodrip-printing on a single substrate can interlace multiple arrays to yield a palette of emissions, without crosstalk between QDs of different colour (Fig. 1g).

As previously demonstrated, electrohydrodynamic nanodrip-printing allows the generation of ordered nano-structures with spatial resolution of 100–200 nm on dry surfaces^18^. As a first step in the adaptation of this technology to TFM and based on the requirements of the cases we studied, we printed monocrystalline nanodisc arrays of custom-made QD dispersions on hydrophilic glass substrates with inter-disc spacing ranging from 0.75 to 3 μm. This resulted in a positioning precision of 30–45 nm, which was almost independent from the inter-disc spacing (Supplementary Fig. 3a). Printing of QDs was subsequently optimized for elastomeric substrates such as CY52-276 and yielded the same precision in nanodisc positioning (Fig. 1h). In general, electrohydrodynamic nanodrip-printing is not limited to this particular silicone and can be equally applied to other silicones. Moreover, with future optimization of the chemistry to ensure stable binding of QDs to polyacrylamide, a cTFM substrate based on a hydrogel could be envisioned (Supplementary Fig. 4).

The achieved printing accuracy (Fig. 1h) is a key enabler in omitting cell removal for the acquisition of a reference image (Fig. 1b). An *in silico* analysis based on the defined material model (Fig. 1d) revealed that with a printing error of 35 nm the resulting noise in the calculated surface tractions increases from 100 to 500 Pa as the inter-disc spacing decreases from 3 to 0.75 μm (Supplementary Fig. 3b,c). We therefore applied monochromatic arrays featuring inter-disc spacing of 1.5 μm to single cells and of 3 μm to cell monolayers as an ideal trade-off between spatial resolution and force sensitivity.

**Figure 2:**
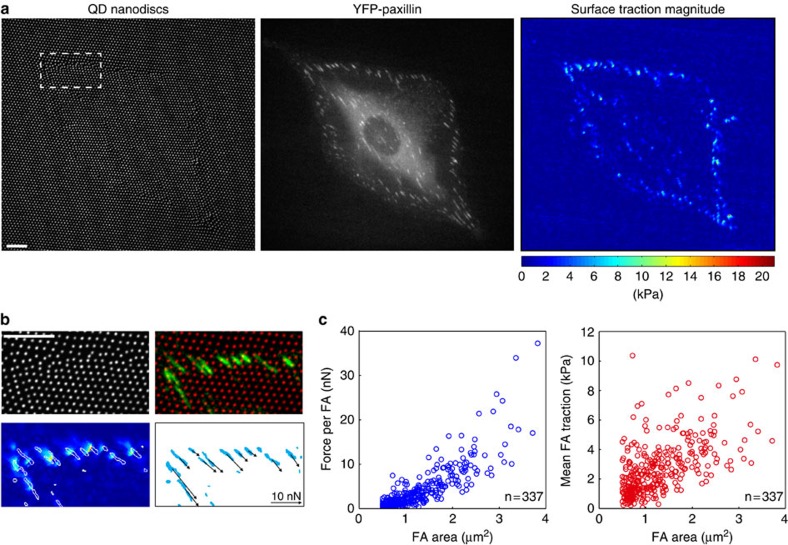
High-resolution detection of forces exerted at focal adhesions. (**a**) REF-Pax cell on cTFM substrate with red QD nanodiscs (spacing: 1.5 μm). Scale bar, 10 μm. Reconstructed surface tractions peaks are located at the cell circumference. (**b**) Surface traction peaks and focal adhesions colocalize. Scale bar, 10 μm. The lower right panel depicts the magnitude and direction of forces exerted by individual focal adhesions. (**c**) Force and traction exerted by individual focal adhesions (only focal adhesions with an area >0.5 μm^2^ were analysed). Data from *n*=337 focal adhesions detected in four cells from independent experiments via integration of traction stress over focal adhesion area.

**Figure 3:**
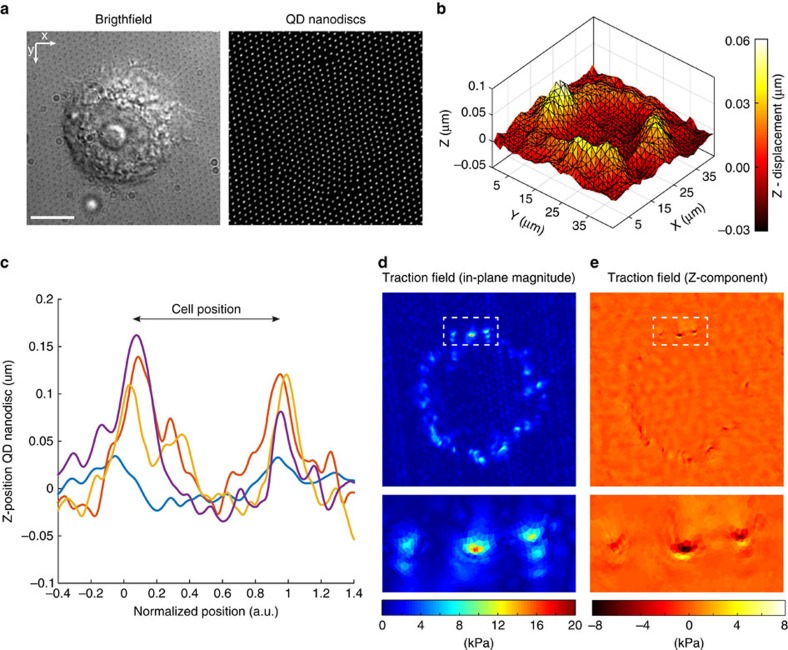
Detection of out-of-plane forces. (**a**) Spreading MCF10A cell on cTFM substrate with red QD nanodiscs (spacing: 1.5 μm). Scale bar, 10 μm. (**b**) Smoothed 3D surface plot of the area underneath the cell in **a**. For traction force reconstruction, the unsmoothed displacements in *Z*-direction are taken into account. (**c**) *Z*-profile of the substrate obtained from line scans underneath four independent cells. Cell dimensions were normalized so that cell outlines reach from 0 to 1. (**d**) Overall traction magnitude of the cell in **a**. (**e**) Out-of-plane (*Z*-) components of the traction field for the cell in **a**.

**Figure 4:**
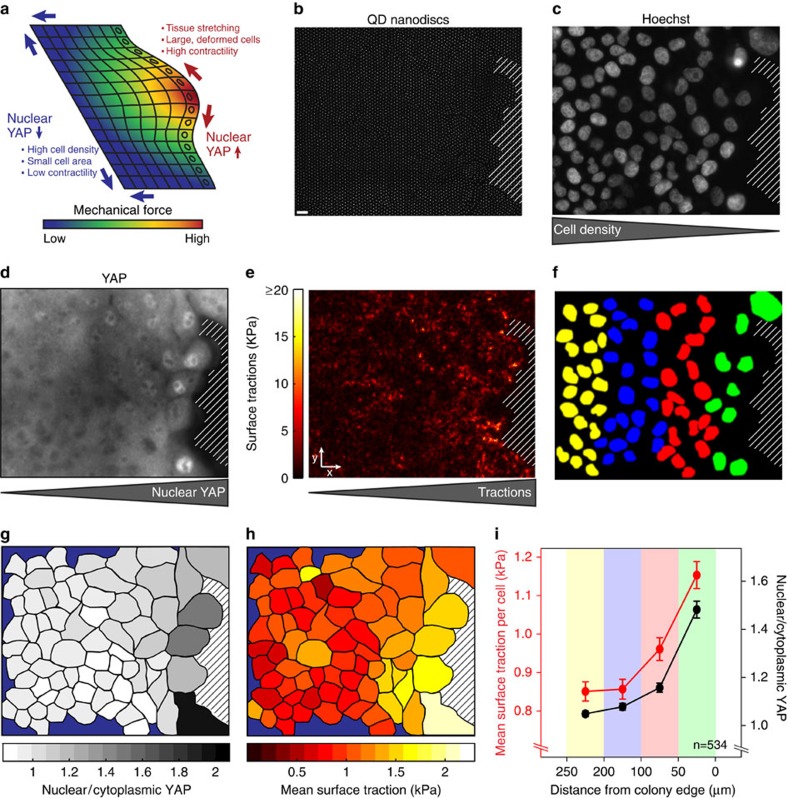
Correlative traction force microscopy. (**a**) Current model of YAP activation in epithelial monolayers (modified from ref. 47). (**b**) Edge of a colony of epithelial MCF10A cells seeded on a cTFM substrate with red QD nanodiscs (spacing: 3 μm). After live imaging of the fluorescent QD nanodiscs cells were immediately fixed and processed for immunostaining. Scale bar, 10 μm. (**c**) Nuclei and decreasing cell density along the *x* axis. (**d**) Immunostaining for the transcription factor YAP. (**e**) Surface traction exerted by the MCF10A monolayer. (**f**) Binning of nuclei into 50 μm regions of interest (ROIs) perpendicular to the colony edge. (**g**) On the basis of segmentation of the brightfield image, individual cell outlines were defined and nuclear/cytoplasmic YAP was quantified for each cell. (**h**) Mean surface traction per cell. Blue region corresponds to cells with cropped nuclei. Therefore no nuclear/cytoplasmic YAP could be quantified and cells were excluded. Hatched area corresponds to a region devoid of cells. (**i**) Cells from four independent colony edges were binned in four ROIs (as defined in **f**) and surface traction per cell and nuclear/cytoplasmic YAP ratio were determined. *n*=number of analysed cells; error: s.e.m.

After coating with fibronectin, adhering cells generate deformations of the substrate, which are captured as distortions of the fluorescent nanodisc array. A host of different cell types including, but not limited to, rat embryo fibroblasts (REF-Pax^32^), HeLa cells, human umbilical vein endothelial cells and MCF10A mammary epithelial cells proved compatible with the cTFM platform and induce dynamic deformations of the substrate to different extents (Figs 2a, 3a and 4b, and Supplementary Movies 2 and 3). In addition, the cTFM platform is fully compatible with biological applications, as no cytotoxicity is detected^33^ (Supplementary Fig. 5).

On imaging of the cTFM platform in confocal or wide-field fluorescent microscopy, the position of individual nanodiscs on the deformed substrate is determined with sub-pixel resolution by calculating their weighted centroid (Fig. 1i). An analysis using *in silico* generated images (for details see Methods) revealed that this detection algorithm has a precision of 4–8 nm depending on the amount of imaging noise (Supplementary Fig. 6a,b). A custom-made algorithm is next utilized to construct the triangular mesh by identifying the six original neighbours for each individual QD nanodisc (Fig. 1i; see Methods and Supplementary Methods for details). The corresponding (load-free) regular arrangement of the disc array is then computationally reconstructed by relaxing the triangular mesh back to an equilateral configuration (see Methods). This procedure tracks individual nanodiscs and allows to reliably extract displacement vectors (Fig. 1i, Supplementary Fig. 6c–e, and Supplementary Movies 3 and 4). Analysis of the *in silico* generated images showed that the reconstruction algorithm is precise, resulting in positioning errors ≤30 nm (Supplementary Fig. 6b), which are within the limit of our printing precision. On the basis of the displacement vectors obtained from the reconstruction algorithm, the kinematic boundary conditions of each node on the surface of a finite element mesh are defined by means of interpolation with radial basis functions (see Methods). Surface tractions, defined in the deformed configuration (Cauchy tractions), are finally obtained using nonlinear finite element analysis (FEA, Fig. 1i, for details see Methods). The FEA accounts for both the nonlinear material properties of the substrate and the inherent geometrical nonlinearity associated with large deformations observed in cTFM experiments, typically with strains larger than 100%. For a 9:10 CY52-276 mixing ratio, this method provides a sensitivity of stress detection of 200 Pa (Supplementary Fig. 7). Owing to the nanometre precision in the printing of nanodiscs and in the detection of their displacement, the calculation of generating traction fields does not require a regularization step, circumventing this difficulty present in particle image velocimetry (PIV)-based TFM procedures. cTFM is thus free from subjective parameterizations, which are prone to the underestimation of tractions in regions of the substrate where high deformations are detected^34^,^35^.

### High-resolution force detection at single focal adhesions

To validate our force detection system we plated REF-52 cells stably expressing YFP-Paxillin (REF-Pax) on a red-emitting cTFM platform. This allowed for simultaneous analysis of substrate deformations and imaging of cell adhesion structures (Fig. 2a). The reconstructed traction peaks were mainly located at the cell perimeter, with nearly exact correspondence to focal adhesions (Fig. 2b). The integral of surface tractions (ranging from 2 to 20 kPa; Fig. 2b) over the area defined by the Paxillin signal yields corresponding values for forces exerted per focal adhesion, which range from 1 to 30 nN (Fig. 2b,c). These results are in good agreement with values obtained on similar cells via classical continuum approaches^36^,^37^, elastic PDMS pillars^6^,^7^,^38^ or DNA-based force sensors^17^. Moreover, compared with other continuum reference-free methods based on lithographic or micro-patterning approaches^14^,^15^,^16^, cTFM features significant advantages: electrohydrodynamic nanodrip-printing enhances spatial resolution, while simultaneously reducing the size of the fluorescent marker below optical resolution, allowing for a highly precise and robust spatial detection. In addition, cTFM provides a homogenously adhesive substrate with height differences ≤30 nm (compared with 300 nm; ref. 16), which is below the threshold at which substrate topography interferes with cells^31^.

### Out-of-plane force detection

Confocality of individual QD nanodiscs and of the nanodrip-printed disc array enables the monitoring of substrate deformations in all directions and with all fluorescent microscopy setups. Cells on planar substrates exert forces in all dimensions, with normal (out-of-plane) traction components typically being lower than in-plane ones^39^. Detection of these out-of-plane force components has led to fundamental findings in the field of cell adhesion biology^39^,^40^. To demonstrate the capability of cTFM in terms of out-of-plane force detection, we obtained three-dimensional (3D) image stacks of the fluorescent nanodisc array, deformed by spreading MCF10A cells. Intensity profiling along the *Z* axis was used to detect normal displacements (see Methods for details). We found that MCF10A cells generate significant normal displacements on spreading on the substrate, well above our vertical detection limit (Fig. 3a–c and Supplementary Fig. 8). In line with previous work^39^,^40^ cells pull upwards at the leading edge, and tend to push downwards towards the cell centre (Fig. 3b,c). The detected vertical displacements are included in the reconstruction algorithm to calculate the volumetric traction field at the surface of the substrate, as well as corresponding principal traction vectors (Fig. 3d,e). The ratio of out-of-plane and in-plane components of the tractions ranges from 0.1 to 0.5, illustrating the substantial magnitude of normal tractions^40^. Our results demonstrate that the cTFM platform is capable of separate detection of in-plane and out-of-plane components of cell tractions with high resolution. This is a remarkable advantage over other available discrete or continuum reference-free methods. PDMS pillars^6^,^7^ have lower spatial resolution and only detect in-plane tractions, whereas molecular force sensors^17^, despite their very high spatial resolution, cannot discriminate force direction. In addition, no out-of-plane force detection has been demonstrated using continuum reference-free methods^14^,^15^,^16^, to the best of our knowledge. cTFM is thus the first reference-free TFM method based on a two-dimensional substrate capable of detecting cellular tractions exerted in all directions.

### Correlative cTFM

The cTFM platform enables the direct correlative overlap of high-resolution continuous traction force fields with spatial localization of proteins via immunofluorescence methods. This capability has so far been limited to micropillar substrates^7^,^41^,^42^,^43^, which geometrically constrain cell adhesion and might alter cell behaviour. cTFM thus expands the portfolio of possibilities, in addition to the use of live-cell reporters. Such capability is of unique value when addressing post-translational protein modifications such as site-specific phosphorylation, where, to the best of our knowledge, no live reporter exists that detects the presence of specific phospho-groups on target substrates. We illustrate this potential visualizing the phosphorylation pattern of the focal adhesion protein Paxillin^44^ (Supplementary Fig. 9).

To further showcase the capability of cTFM, we applied it to a recently discovered molecular pathway implicated in the transduction of mechanical signals in epithelial monolayers. Signalling pathways activated by mechanical stimuli can drive whole sheet responses, while being active only in a subset of cells at specific locations within the cell layer, such as wound edges, replicative foci and others^45^. Perhaps the most prominent example is the mechano-sensitive pathway of Hippo^46^, which controls the nuclear translocation of the transcription factor YAP (yes-associated protein). Gradients of YAP activation, commonly revealed by immunostaining, develop from the edge of the epithelium to the rear positions. Therefore, cells at the boundary retrieve positional information, which is assumed to be functionally linked with forces, including both intercellular and cell–extracellular (ECM) traction forces^47^ (Fig. 4a). We applied cTFM to directly correlate epithelial force generation and nuclear translocation of YAP. To this end, MCF10A mammary epithelial cells were seeded in dense clusters on red cTFM substrates. At the edge of the epithelial colony we detected traction forces within the cell monolayer (Fig. 4b), followed by immediate fixation and immunofluorescence processing for YAP and Hoechst (Fig. 4c,d). As suggested by previous literature, we found that cells in the proximity of the free, migrating edge of the colony display a strong correlation between increased nuclear YAP signal and the magnitude of surface tractions^46^,^47^ (Fig. 4d–i). We are not aware of any other studies and methods that have directly (for the same cells) and quantitatively correlated YAP localization with the magnitude of traction forces. Therefore, cTFM fully preserves the information on cell position and identity during downstream processing. This information enables the generation of colocalization maps and allows for spatially resolved quantitative analysis of traction forces and cellular signalling (Fig. 4f–i). Further, the data show that cTFM is not limited to single-cell analysis but can be applied to cell cohorts and collectively migrating cell monolayers.

## Discussion

The presented results establish cTFM as an easy-to-use, high-resolution force sensing method markedly advancing the state of the art over current TFM approaches. The highly deformable silicone-based substrate combined with a nanodrip-printed monocrystalline array of fluorescent landmarks constitutes an optically transparent, continuum environment, which can be adapted to a broad palette of biological applications. The selected two-component silicone allows for traction measurements over a wide range of physiological forces with straightforward fabrication requirements. For the cells and applications described here, the used 9:10 mixing ratio proved to be suitable in terms of magnitude of displacements and resulting material strains. Cell-induced displacements were well above nanodrip-printing noise (≈30 nm), while the resulting strains in the substrate were within the regime analysed in the mechanical tests (Fig. 1d). For other cells or applications, fabrication and characterization of further CY52-276 mixing ratios can expand the force detection range of the cTFM platform in the future.

The well-defined and highly stable QD emission is homogenous across the nanodisc-lattice and can be tailored over a large palette of colours. Similar to conventional TFM, overlays of crystalline array with multiple colours could be used in the future to further increase spatial resolution of the cTFM platform^13^. The use of the nanodrip-printing technology enables the deployment of QDs into sub-resolution nanodiscs arranged in a confocal and crystalline triangular pattern. Therefore, it yields a substrate for cells, which is homogeneously adhesive and devoid of any intrusive topography. Finally, the corresponding fully nonlinear analysis enable application of the cTFM platform to highly deformable substrate materials and large strain problems, beyond the validity of linear methods^3^.

The synergistic effect of the above-mentioned technologies enables a one-step-imaging process, which directly provides all necessary information to compute the intensity and direction of in- and out-of-plane traction forces produced by cells. This substantially simplifies the experimental workflow and supports a rapid and high-throughput analysis. Importantly, it removes the requirement for cell detachment and acquisition of a load-free reference image, enabling correlative visualization of traction forces and cellular signalling activity, and rendering cTFM a significant methodological advancement in cell force determination and activity for the biological and medical communities.

## Methods

### Cell culture

HeLa cells (American Type Culture Collection; ATCC) and rat embryonic fibroblasts stably expressing YFP-Paxillin (REF-Pax^32^) were grown in high-glucose Dulbecco's modified Eagle's medium (DMEM) supplemented with 2 mM L-Glutamine, 10% FBS, 1% penicillin/streptomycin (all Sigma-Aldrich). Human Umbilical Vein Endothelial Cells (HUVECs, Life Technologies) were grown in M200 medium with Low Serum Growth Supplement (all Life Technologies). MCF10A cells (G. Scita, IFOM Milan, Italy) were grown in Dulbecco's modified Eagle's medium /F12 medium (Life Technologies) supplemented with 10% horse serum, 1% penicillin/streptomycin, 0.5 mg ml^−1^ hydrocortisone, 10 μg ml^−1^ insulin, 20 ng ml^−1^ epidermal growth factor (all Sigma-Aldrich) and 100 ng ml^−1^ Cholera toxin (List Biological Laboratories). All cells were cultured in a humid atmosphere containing 5% CO_2_ at 37 °C.

PC12 cells (ATCC) were grown in RPMI-1640 medium supplemented with 10% horse serum, 5% FBS, 2 mM L-glutamine and 1% penicillin/streptomycin (all Sigma-Aldrich). Details on the cytotoxicity test are described elsewhere^33^. Briefly, PC12 cells were grown for 4 days in RPMI-1640 medium supplemented with 2% FBS, 2mM L-glutamine, 1% penicillin/streptomycin and 100 ng ml^−1^ nerve growth factor (induction medium), followed by measuring the mean neurite length.

### Substrate fabrication and nanodrip-printing

The two components of CY52-276 polydimethylsiloxane (Dow Corning) and 0.05% (v v^−1^) poly(dimethylsiloxane-*b*-ethylene oxide) (Polysciences) were mixed thoroughly at the desired ratio for 5 min, degased for 2 min and spin-coated on 170 μm thick coverslips for 1 min at 1,500 r.p.m. The silicone was then cured at 70 °C for 30 min. Afterwards the substrates were kept at all stages in a clean, dust-free and dry environment to prevent fouling. With respect to ageing properties of polydimethylsiloxanes^30^, we always used CY52-276 samples of the same age for experiments (2–3 weeks).

The red core-shell-shell CdSe-CdS-ZnS QDs with an emission peak at 627 nm were synthesized following a published recipe^48^. The detailed recipe, as well as recipes for the blue and green QDs can be found in the Supplementary Methods. The QDs were transferred from hexane dispersions to tetradecane for the printing process. To ensure a reproducible printing process and reduce clogging at the printing nozzle, the optical density of the dispersion at the first absorption peak was adjusted to 0.5 for a 1 mm path and then further diluted 1:1 in tetradecane. The deposition precision was further increased by adding 5% vol of octanethiol-capped gold nanoparticles^49^ to the QD ink from a tetradecane dispersion with an optical density of 5 for a 1 mm path. These gold nanoparticles enable a more stable printing process, leading to a reproducible droplet ejection and thus a higher placement precision, but do not adversely affect the fluorescence.

The QDs were deposited on the substrate by electrohydrodynamic nanodrip-printing, details of which have been published elsewhere^18^,^19^,^20^,^21^. Briefly, the substrate is placed on a conducting grounded plate. A gold-coated glass capillary with an opening diameter of 1–1.5 μm is filled with the QD dispersion and brought within 5 μm of the substrate using a piezoelectric stage with nanometre precision. By applying voltage pulses between the nozzle and the grounded plate, nanoscale droplets with a diameter of 50–100 nm are rapidly ejected from the apex of a larger meniscus formed at the nozzle exit with frequencies of 100–200 Hz. The droplets land softly on the substrate (no splashing or sizable spreading) and the tetradecane evaporates before the arrival of the next droplet, leaving behind only the nanoparticle content. To print one nanodisc of the triangular array, d.c. voltages of 200–250 V are applied for 70 ms. In this manner, the QDs of several nanodroplets land at the same location each time and form collectively one brightly emitting disc at a well-defined position. Arbitrary patterns can be created moving the substrate with the piezoelectric stage. Voltage, pulse length and stage position are controlled using a custom-built control unit. The electrohydrodynamic nanodrip-printing technology is freely available for laboratory research and, in addition, can be commercially obtained through an ETH Zurich spin-off company (http://www.scrona.ch).

### Mechanical characterization of the substrate

The mechanical properties of the substrate were evaluated for thin cast samples loaded in different modes. Uniaxial-tension tests were performed on a tensile testing set-up mounted on a MTS 793 testing rig (MTS Systems, Eden Prairie, USA). Equibiaxial deformation behaviour of the material was characterized on a custom-built inflation set-up^30^. All measurements used for the evaluation of the elastic properties were performed at low strain rate (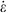
=0.3% s^−1^) and within the range of deformation expected to occur during cTFM (up to 175% strain). Strain was quantified by tracking optical features on the sample surface using a CCD (charge-coupled device) camera (Pike 100B Allied Vision Technologies GmbH) and a customized tracking algorithm^30^. The thickness of each sample was determined post testing by optical measurements of the cross-section of sliced samples in a microscope (LSM 5 PASCAL, Zeiss). The stress–strain data of the tests were used to fit the parameters of a hyperelastic Ogden material^50^ with two terms (Table 1).

### ECM coating and cell seeding

Before coating with the ECM protein fibronectin, substrates were incubated in a custom-built vacuum oven at 90 °C for 1 h, washed 1 min in methanol and incubated for another 2 h in the vacuum oven to remove ligands and anneal QDs to the silicone. Substrates were then glued in 35 mm Petri dishes. For coating 50 μg ml^−1^ fibronectin (Life Technologies) in PBS was applied to the substrates for 1 h at room temperature. Substrates were washed twice with PBS before applying medium and seeding of cells at desired concentrations.

### Live-cell imaging

Cells were allowed to spread from at least 3 h till overnight before imaging (except otherwise noted) using an inverted Nikon-Ti wide-field microscope equipped with an Orca R-2 CCD camera (Hamamatsu Photonics, Japan) or an iXon Ultra 888 EMCCD (Andor, UK). Temperature, CO_2_ and humidity were controlled during imaging using an incubation chamber (Life Imaging Services, Switzerland). Images were collected using a × 60 Plan Apo λ 1.40 Oil or and × 60 Plan Apo VC 1.20 water objective. Focal drift during the experiments was avoided using the autofocus system of the microscope.

### Image analysis

The pointspread function of individual 50 nm fluorescent beads on polyacrylamide and CY52-276 silicone was distilled from 3D image stacks acquired with conventional wide-field fluorescent microscopy using Huygens software (Scientific Volume Imaging).

Focal adhesion structures were segmented based on a previously described method^51^, except that the mathematical exponential step was omitted. Briefly, on background subtraction, the local contrast of the image was enhanced via Contrast Limited Adaptive Histogram Equalization. After applying a Laplacian of Gaussian filter, the image was manually tresholded and particles were analysed. Only focal adhesions with a size >0.5 μm^2^ were considered for the analysis.

### QD nanodisc detection and meshing

Traction evaluation starts with the detection of the *XY* positions of the QD nanodiscs. A threshold was applied to the image and the connected pixel islands were identified as the QD nanodiscs. The exact position of the QD nanodisc was calculated by taking the weighted centroid of the grey scale value of the connected pixel islands. To evaluate the deformation along the *Z* axis, 3D image stacks were acquired (⩾30 slices, spacing 100 μm). The spot detection function of Imaris (Bitplane) was used to detect the *Z* position of the QD nanodiscs (spot size: *XY*, 0.717 μm; *Z*, 1.434 μm). By fitting a plane through all points (Matlab, Mathworks) and taking the difference between the plane and the measured *Z* coordinates, a tilt correction was applied to filter for skewness between the sample and the focal plane.

The next step in the analysis procedure was the construction of the triangular mesh with a two-step custom-built algorithm, achieved by identifying the original six neighbours for each individual QD nanodisc (for a detailed description see Supplementary Information). Briefly, in a first step the regions of the image with no or low distortion were identified. In the non- or low-deformed configuration each QD nanodisc has exactly six neighbouring QD nanodiscs at equal distances and forming angles of 60°. Each QD nanodisc is locally checked for this condition and connected to its six neighbours only if this condition holds, up to a tolerance of ≤250 nm and 10° to account for small deformations. At the end of this analysis, the regular parts of the image are meshed, that is, connected to an array of triangles, whereas the QD nanodiscs in regions with high deformation remain unmeshed, forming voids in the mesh (Supplementary Fig. 10). The boundary of each void gives sufficient information to generate a perfect mesh with the same geometry as the void and has the same number of vertices as there are unconnected QD nanodiscs in void. The algorithm then computes the optimal assignment of vertices of the perfect mesh to positions of QD nanodiscs by minimizing the overall mesh distortion.

### Reference configuration reconstruction

The regular initial arrangement of the QD nanodiscs permits the reconstruction of the reference configuration without acquiring a load-free image, exploiting the fact that the reference distance between two neighbouring nanodiscs is known (*L*_0_) and equal for all nanodiscs. For this procedure, the QD nanodiscs are idealized as point masses and connected to their neighbours (identified in the previous step, see paragraph above) with pre-stretched springs of reference length *L*_0_. Minimization of the potential energy relaxes the deformed spring-mass network towards the steady-state solution, which corresponds to the stress-free configuration where all the springs have length *L*_0_. To stabilize the computation an inconsequential damping component is added and the differential equation describing the dynamics of the damped mass-spring network is solved explicitly using a fourth-order Runge–Kutta integration scheme.

### Nonlinear FEA-based traction reconstruction

To account for both large deformations and material nonlinearity, a custom high-resolution nonlinear TFM framework was developed based on Python scripts (Python Software Foundation) and a commercial finite element code (Abaqus, Dassault Systèmes). The framework contains three principal steps: (I) model creation and finite element meshing; (II) application of displacement boundary conditions; and (III) computation of the traction stress field.

In the first step (I), the finite element geometry models a cuboid section of the substrate, whose height is equal to the actual substrate. In-plane dimensions are chosen larger than the analysed cell, so that the border zones of the section contain undeformed regions. An adaptive meshing algorithm was implemented that selects the QD nanodiscs with high displacement magnitude and automatically meshes with linear hybrid tetrahedral elements the corresponding regions with a fine mesh (elements five times smaller than QD nanodisc interspacing), whereas the other regions are meshed by elements of increasing size (up to QD nanodisc spacing). Material properties were defined based on the nearly incompressible implementation of the Ogden model (Poisson's ratio 0.49) provided by the software, with parameters given in Table 1 obtained by fitting the experimental data (Fig. 1d).

To determine the displacement boundary conditions (II), an interpolation based on thin-plate spline radial basis functions^52^ is applied to prescribe the displacement at each node of the finite element mesh on the cell sided surface of the substrate. This is necessary, since image analysis provides the displacements only at the position of the QDs. To account for the bonding of the substrate to the glass coverslip and the embedding of the model into a larger portion of the substrate, the degrees of freedom of nodes on the lateral and bottom sides of the section were fixed.

Finally (III), the implicit FEA solver is used to compute the strain and stress states in the substrate, taking into account the applied boundary conditions and the nonlinear material behaviour. The computed solution includes reaction forces for all the nodes that are constrained by displacement boundary conditions. These forces are subsequently related to the deformed surface area of the elements, finally providing the traction field induced by the cell. It is important to note that, as required in the case of large deformations, tractions are defined in the deformed configuration (Cauchy tractions).

### Immunofluorescence

Cells were fixed with 3% paraformaldehyde in PBS for 10 min at room temperature and permeabilized via incubation in 3% paraformaldehyde with 0.1% Triton in PBS for 5 min. After blocking with 5% BSA in PBS for 2 h, samples were incubated at 4 °C overnight with a monoclonal primary antibody against YAP (Cell Signaling (D8H1X), dilution: 1:300) or phosphorylated Paxillin (Tyr118, Cell Signaling (#2541), dilution: 1:300). Alexa Fluor 647 Chicken anti-Rabbit (Thermofischer (#A-21443)) was used 1:200 as secondary antibody for 1 h at room temperature. For staining of nuclei, Hoechst was added at 10 μg ml^−1^ during a washing step.

### *In silico* image generation

A synthetic image that simulates a real image acquired in the microscope was created with the following procedure^53^. First, the positions of the QD nanodiscs in perfectly regular arrays and arrays with positioning errors of 35 nm (recapitulating the printing error) were determined using MATLAB. Then, in some regions these positions were displaced to a maximum of 1.5 μm to simulate cell tractions. A synthetic image was obtained by creating Gaussians with s.d. of 0.2 μm at the exact positions of the nanodiscs, to simulate the signal convolution of the microscope. Finally, Gaussian noise with s.d. of 10 was added to the synthetic image in ImageJ to mimic imaging noise.

### Data analysis and statistics

Data were analysed, tested for statistical significance, fitted and visualized using R or MATLAB (The MathWorks). No statistical method was used to predetermine sample size. No estimation of variance was performed. The Shapiro–Wilk test was used to test for normality of data. For non-normal distributed data, Mann–Whitney *U*-test was performed.

### Data availability

cTFM software featuring algorithms for QD nanodisc detection, meshing, reference configuration estimation and displacement reconstruction, and surface traction evaluation is available as Supplementary Software (cTFM_Package_NatComm.zip and cTFM_Examples_NatComm.zip) at https://dx.doi.org/10.6084/m9.figshare.3493685.v1. cTFM_Package_NatComm.zip contains the software package used to analyse cTFM images (including documentation). cTFM_Examples_NatComm.zip contains example images to be analysed with the cTFM software. Further relevant data are available from corresponding authors on request.

## Additional information

**How to cite this article:** Bergert, M. *et al*. Confocal reference free traction force microscopy. *Nat. Commun.* 7:12814 doi: 10.1038/ncomms12814 (2016).

## Supplementary Material

Supplementary Information Supplementary Figures 1-10, Supplementary Methods and Supplementary References

Supplementary Movie 1 Electrohydrodynamic nanodrip-printing of fluorescent quantum dot nanodiscs. Scale bar: 5 μm

Supplementary Movie 2 Migrating HUVEC on red cTFM platform (inter-disc spacing: 1.5 μm). Cell und nucleus outline (based on brightfield image) were superimposed. Scale bar: 10 μm.

Supplementary Movie 3 Relaxation of the elastic silicone upon cell removal. HeLa cells were seeded on a red cTFM platform (inter-disc spacing: 1.5 μm). Upon imaging, cells were detached from the substrate by adding 0.01% sodium dodecyl sulfate to the medium, followed by imaging the same region again. Scale bar: 10 μm.

Supplementary Movie 4 Mesh relaxation. Reconstruction of the original mesh and QD nanodisc's original positions through relaxation of the deformed mesh. Colorbar: 1 corresponds to maximum deformation of the triangles in the mesh and 0 to perfectly equilateral triangles. Red dots indicate QD nanodiscs whose positions are fixed as boundary for the relaxation.

## Figures and Tables

**Table 1:** Ogden material parameters.

***μ***_**1**_ **(kPa)**	***α***_**1**_ **(−)**	***μ***_**2**_ **(kPa)**	***α***_**2**_ **(−)**
4.073	2.132	0.167	−0.600
