# Supplementary material for: Confocal reference free traction force microscopy
Source: Nat Commun. 2016 Sep 29;7:12814. doi: 10.1038/ncomms12814 (PMC5056408; doi:10.1038/ncomms12814)
Supplement: Supplementary Information — Supplementary Figures 1-10, Supplementary Methods and Supplementary References [file ncomms12814-s1.pdf]

Supplementary Information – "Confocal Reference Free Traction Force Microscopy"

Martin Bergert*[1], Tobias Lendenmann*[1], Manuel Zündel*[2], Alexander E. Ehret[2,3], Daniele Panozzo[4,5], Patrizia Richner[1], David K. Kim[6], Stephan J.P. Kress[6], David J. Norris[6], Olga Sorkine-Hornung[4], Edoardo Mazza[2,3], Dimos Poulikakos[‡1] and Aldo Ferrari[‡1]

* Equal contribution

[‡‡] Corresponding authors: dimos.poulikakos@ethz.ch, aferrari@ethz.ch

1 - ETH Zurich, Laboratory of Thermodynamics in Emerging Technologies, Sonneggstrasse 3, 8092 Zurich, Switzerland
2 - ETH Zurich, Institute for Mechanical Systems, Leonhardstrasse 21, 8092 Zurich, Switzerland
3 - Empa, Swiss Federal Laboratories for Materials Science and Technology, Überlandstrasse 129, 8600 Dübendorf, Switzerland
4 - ETH Zurich, Institute for Visual Computing, Interactive Geometry Lab, Universitätstrasse 6, 8092 Zurich, Switzerland
5 - New York University, Courant Institute of Mathematical Sciences, 719 Broadway, New York, NY 10003, USA
6 - ETH Zurich, Optical Materials Engineering Laboratory, Leonhardstrasse 21, 8092 Zurich, Switzerland

Supplementary Figures

a

CY52-276 A/B mixing ratio [w/w]	Bulk elastic modulus [kPa]	Reference
6:5	1	23
1:1	3	22,24,25
9:10	12.6	Fig. 1d
5:6	18.6	Suppl. Fig. 1f

b

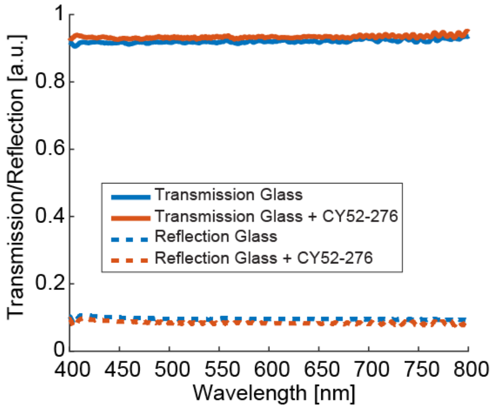

c

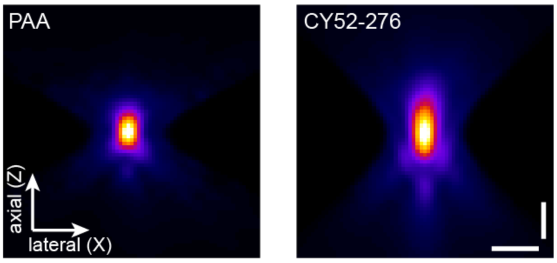

d

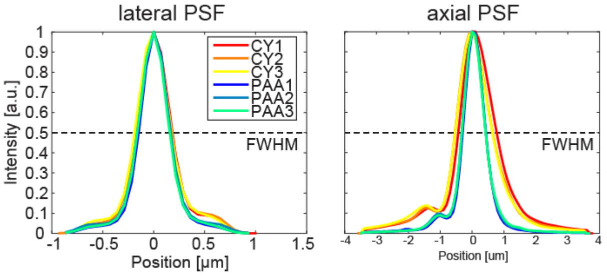

e

sample	FWHM lateral	FWHM axial
PAA	320 ± 8 nm	744 ± 15 nm
CY52-276	363 ± 4 nm	1187 ± 16 nm

f

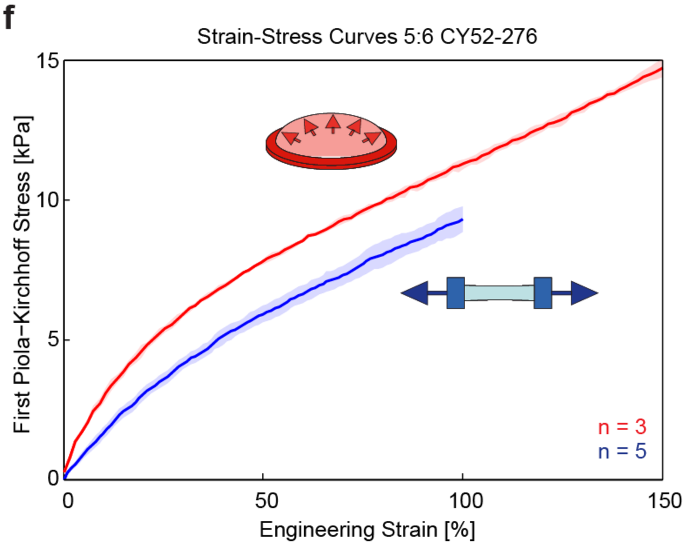

g

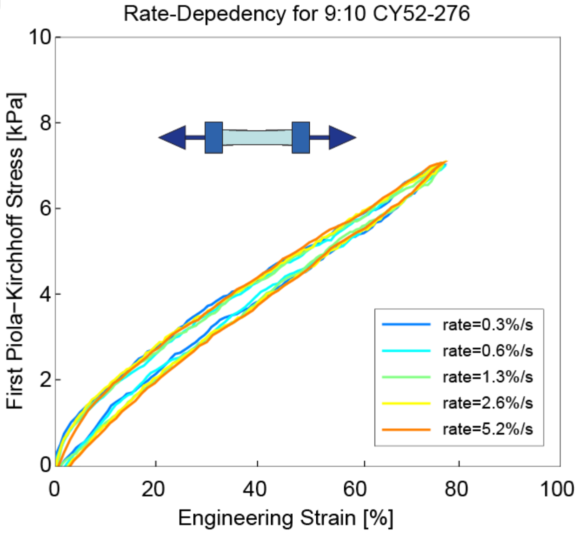

Supplementary Fig. 1: Mechanical and optical properties of CY52-276 silicone.

a - Elastic moduli of CY52-276 silicone for different mixture ratios. b - Transparency measurements of glass and glass coated with a 30 nm thick CY52-276 layer. c - Examples for PSF of 50 nm fluorescent beads on Polyacrylamide and CY52-276 silicone, imaged with conventional widefield fluorescent microscopy. Scale bars: 1 µm. d/e - Lateral and axial extensions of the PSF, determined from 3 independent beads for each condition. Error: standard deviation (s.d.). f - Results of tensile (uniaxial) and inflation (equibiaxial) testing of CY52-276 silicone mixed at a 5:6 ratio. Free sample dimensions 40 mm x 10 mm (uniaxial) and diameter 30 mm (inflation). Thicknesses were in the range

from 0.5 mm-0.75 mm; Exact thickness was measured for each sample independently (see Methods). Shaded area: standard deviation (s.d.); $n_{uniaxial}$=5, $n_{equibiaxial}$=3 samples per test. g - Result of a cyclic uniaxial strain rate sweep test with CY52-276 silicone mixed at 9:10 ratio.

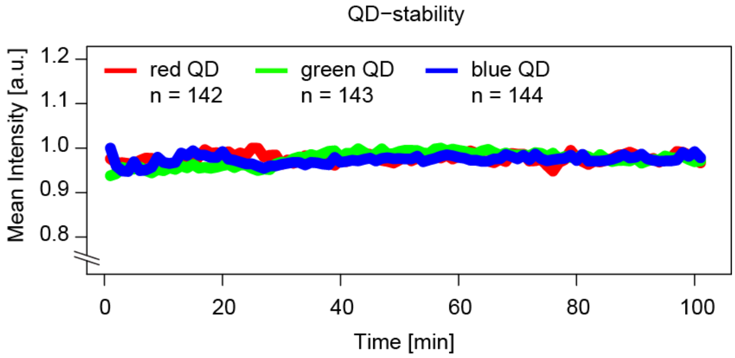

Supplementary Fig. 2: Stability of QDs.

Red, green and blue QD nanodiscs were printed on elastic silicone, post treated and immersed in cell culture medium for 30 min. Imaging of the fluorescent array was then performed every 1 min for 100 time steps and mean intensity of QD nanodiscs was measured. Small fluctuations are caused by focal drift during imaging. n: number of analysed QD nanodiscs.

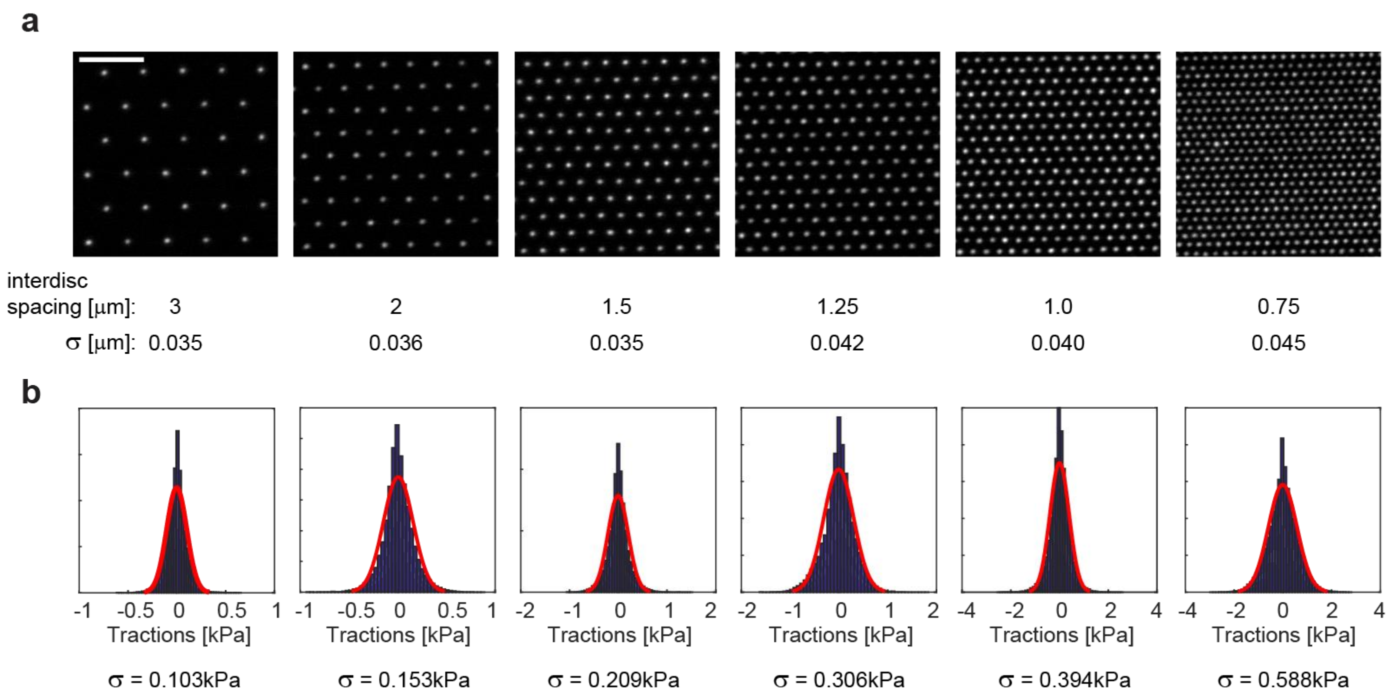

c

Interdisc spacing [µm] (simulated)	Printing noise [µm] (simulated)	Traction sensitivity [Pa]
3	0.035	100
2	0.035	159
1.5	0.035	223
1.25	0.035	279
1.0	0.035	360
0.75	0.035	495

Supplementary Fig. 3: Inter-disc spacing of the cTFM platform.

a - Red QD nanodiscs were printed on glass with inter-disc spacing ranging from 3 to 0.75 µm and the resulting standard deviation (s.d.) for the inter-disc spacing was measured based on fluorescent images (σ). Representative data of n = 2-3 nanodisc arrays. Scale bar: 5 µm. b - Images of the undistorted as-printed arrays (a) were analysed applying the algorithm for estimation of surface tractions (see Methods for details) and the resulting standard deviation (s.d.) of the obtained tractions, which corresponds to the sensitivity in force detection, was determined. c - Simulated undistorted arrays with various inter-disc spacing and a constant printing noise of 35 nm were used to estimate surface tractions and resulted in similar traction force noise levels than the as-printed arrays in a.

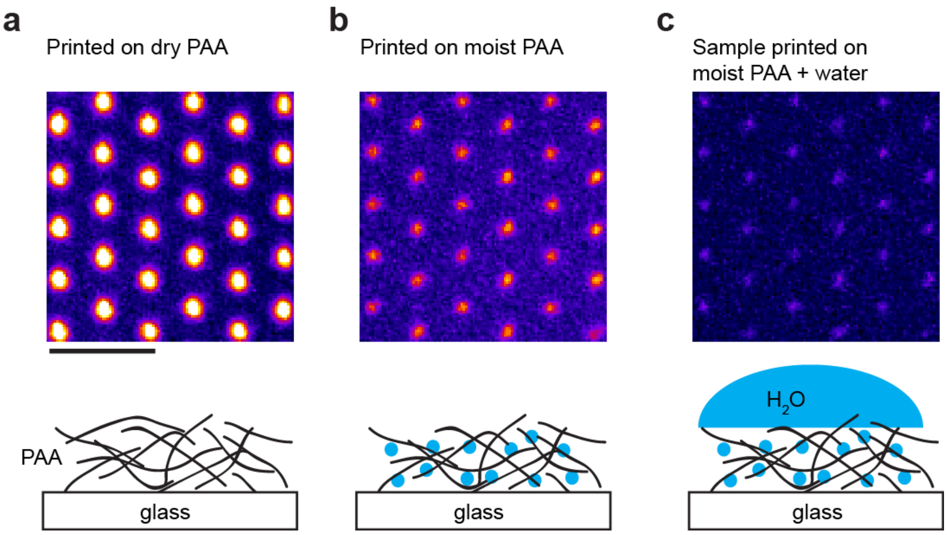

Supplementary Fig. 4: Electrohydrodynamic nanodrip-printing on polyacrylamide substrates.

a - Red QD nanodiscs printed on dry polyacrylamide (PAA) (spacing: 1.5 µm). Scale bar: 3 µm. b - Red QD nanodiscs printed on moist polyacrylamide. c - The sample from B upon immersion in water.

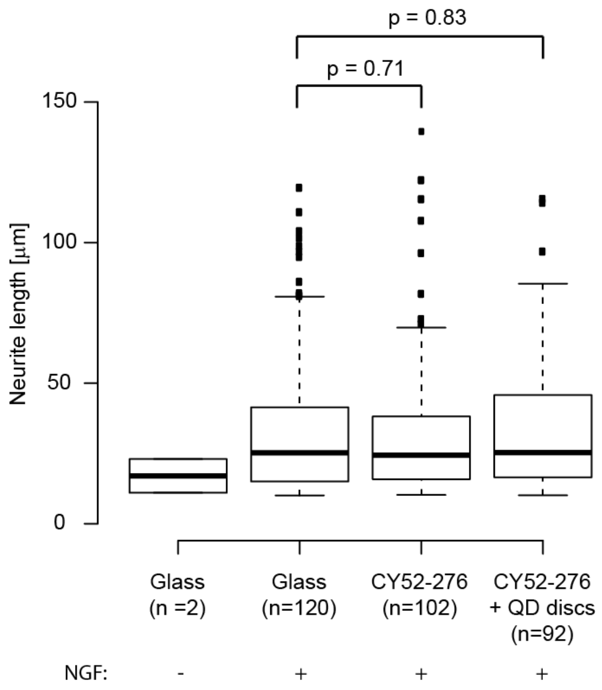

Supplementary Fig. 5: Cytotoxicity assay.

Biocompatibility of the cTFM platform. Plots shows the average length of neurites generated by PC12 cells after 4 days in culture. In the four tested conditions the cells were alternatively plated on control glass substrates, on substrates coated with a thin layer of CY52-276 or on a cTFM platform (CY52-276 + QD nanodiscs). Upon stimulation with NGF, PC12 cells underwent neuronal differentiation with no significant differences between control glass substrates and substrates with CY52-276 or CY52-276 + QD nanodiscs (Mann-Whitney-U-Test). n = number of neurites analyzed on 2 independent substrates for each condition, except for a single substrate for glass without NGF.

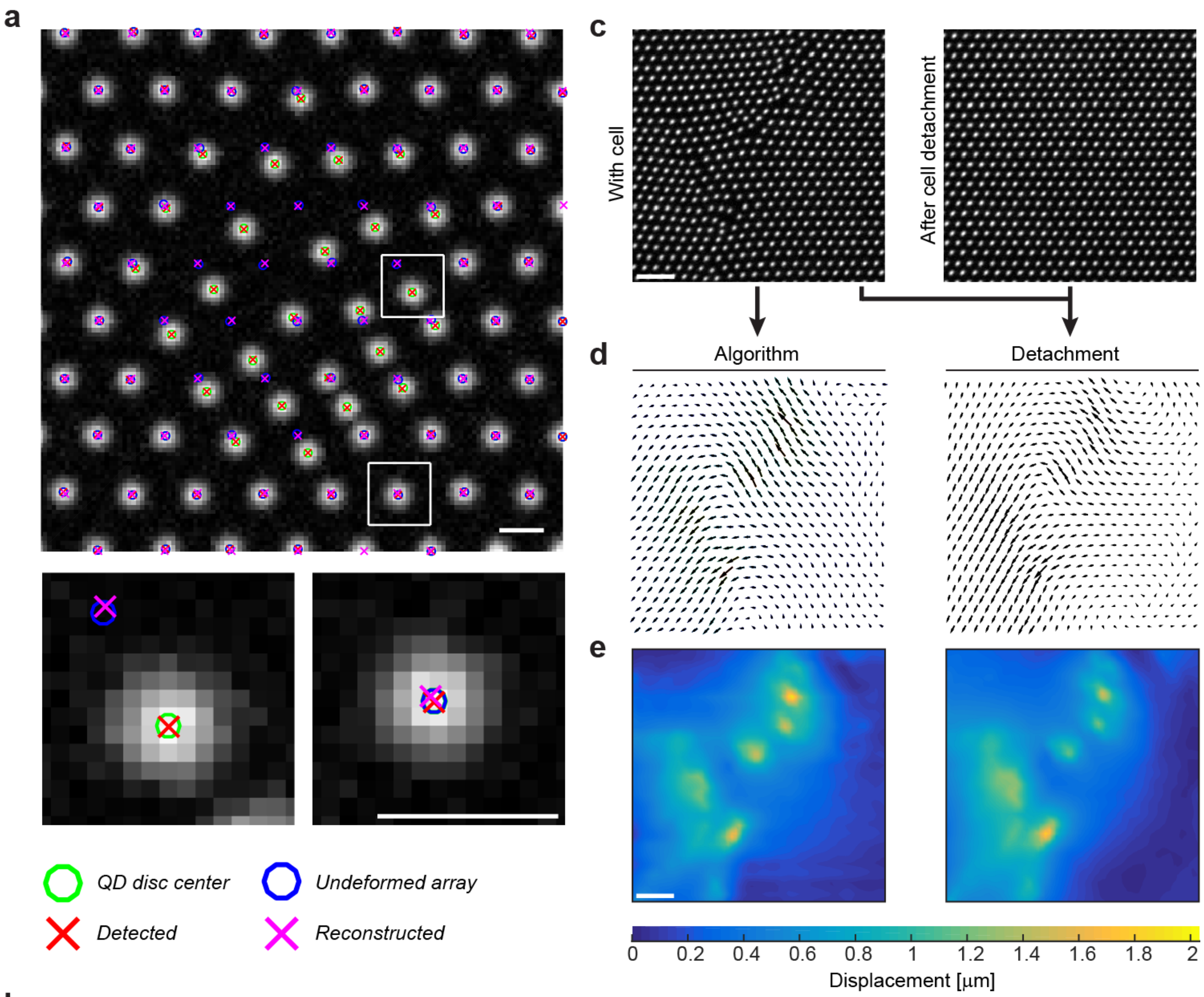

b

Input Data	Detection	Reconstruction	Combined
no noise	4.75 ± 3 nm	0.18 ± 0.16 nm	4 ± 2.4 nm
with 35 nm printing error	6.4 ± 2.7 nm	27.6 ± 17.7 nm	28.8 ± 16 nm
with imaging noise	7.4 ± 3.7 nm	N/A	3 ± 2.4 nm
with 35 nm printing error & imaging noise	8 ± 4.2 nm	N/A	29.1 ± 15.7 nm

Supplementary Fig. 6: Reconstruction of the displacement field.

a - Synthetic images with known imposed initial and displaced positions were used to estimate errors in QD nanodisc detection and displacement field reconstruction. *QD disc center*: imposed center of QD nanodisc, *Detected*: position of QD nanodisc after detection, *Undeformed array*: Position in the initial, undeformed array. *Reconstructed*: Reconstructed initial position of the QD-nanodisc. Scale bars: 1 µm. b - Errors of the detection, the reconstruction and the combined algorithm used to estimate the cTFM displacement fields. Data quantifies mean ± standard deviation (s.d.) of the distances between the following positions: Detection: *QD disc center* - *Detected*; Reconstruction/ Combined: *Undeformed array* - *Reconstructed*. Data for Reconstruction uses *QD disc center* as input,

data for Combined uses *Detected* as input. c - Part of a QD nanodisc array on cTFM substrate with HeLa cells, on the left in distorted configuration and on the right after cell detachment (see Supplementary Movie 3). Scale bar: 5 µm. d - Displacement fields obtained with the reconstruction algorithm (see Methods) and from classical relaxation upon cell detachment. e - Corresponding displacement magnitude maps. Scale bar: 5 µm.

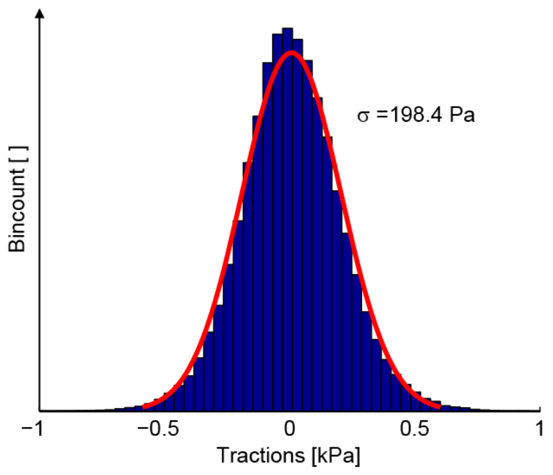

Supplementary Fig. 7: Sensitivity of the cTFM platform.

A representative (n = 5) as-printed array of fluorescent QD nanodiscs on the elastic silicone with a spacing of 1.5 µm and a printing error of $\sigma = 0.033$ µm (Fig. 1h) was used to estimate the sensitivity of the cTFM platform. After the analysis procedure (Fig. 1i), tractions were distributed around 0 kPa with a standard deviation (s.d.) of 0.198 kPa.

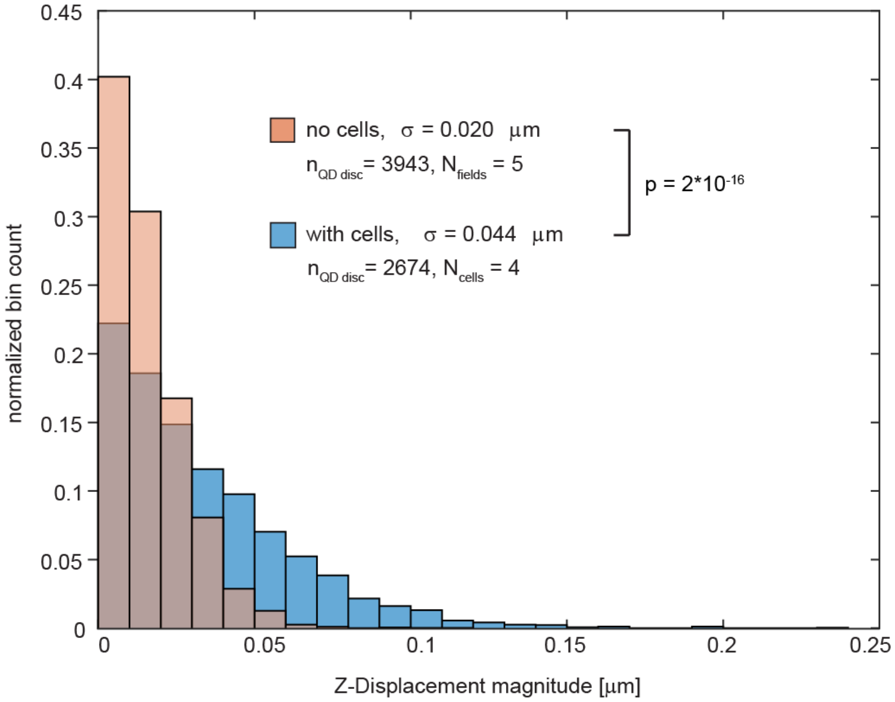

Supplementary Fig. 8: Detection of Z-position of QD nanodiscs.

Flat, undeformed fluorescent nanodisc arrays (n = 5) with spacing of 1.5 µm were imaged as 3D stack using conventional wide-field microscopy (Z-spacing: 100 nm). After evaluation of the Z-position of each QD nanodiscs (see Methods), a standard deviation (s.d.) of about 20 nm was found for the detection of the Z-position, which includes possible minor deviations from perfect flatness of our spin coated CY substrate as well as imaging noise. Upon spreading of MCF10A cells, significantly larger Z-displacements were detected. P-Value: Mann-Whitney-U-Test.

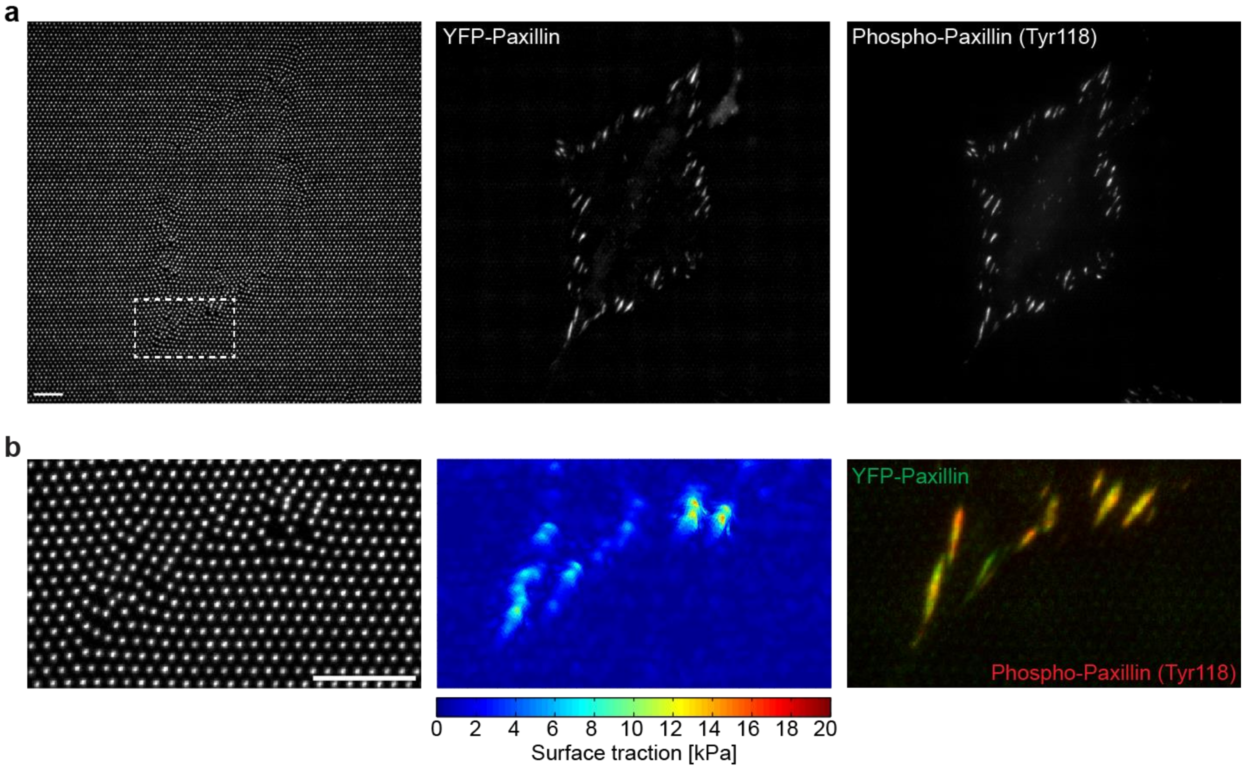

Supplementary Fig. 9: Correlative traction force microscopy using anti-phospho-antibodies.

a - REF-Pax cell on cTFM substrate with red QD nanodiscs (spacing: 1.5 µm). Upon imaging of the QD nanodiscs, the cell was fixed and immunostained for phosphorylated Paxillin. Scale bar: 10 µm. b - Zoom of the region outlined in a. Scale bar: 10 µm.

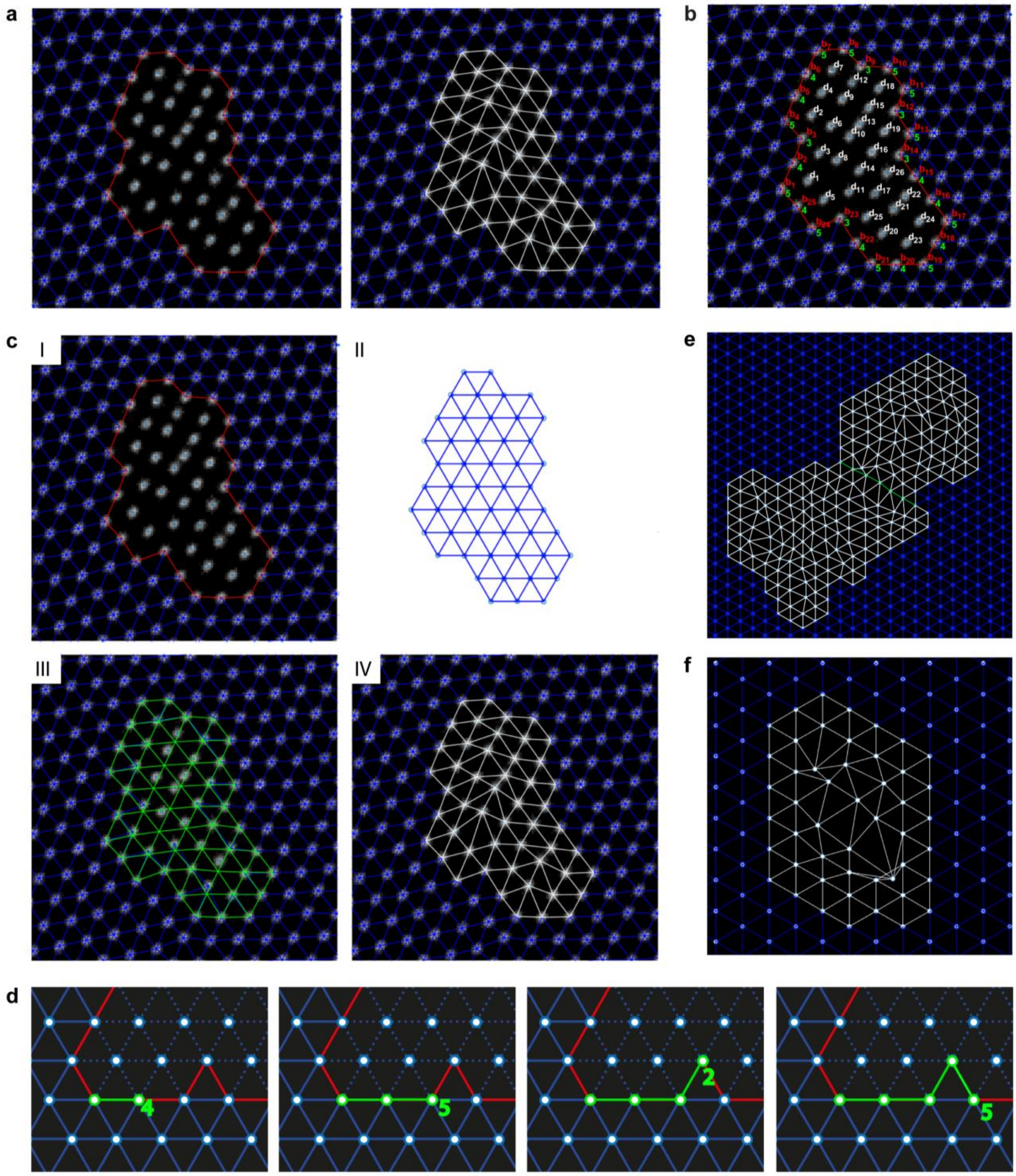

Supplementary Fig. 10: Triangular mesh reconstruction.

a - (left) Only regular regions of the image are meshed (blue) in the first stage of the algorithm. (right) Each void is separately meshed (white) and finally combined to obtain the final mesh. b - Notation for the boundary vertices (red), the number of their connections (green), and internal vertices (white) of a void. c - The three phases of the void meshing pipeline. Unmeshed region (I), perfect flattening (II), boundary fitting (III) and internal snapping (IV). d - Walking along the boundary vertices and taking turns according to the number of connected neighbors. e - Large voids with more than 150 vertices

are slow to process. In these cases, the user shall split the cluster in two parts (green edges), and then process them independently. f - Even in cases with extreme deformations, the algorithm reliably reconstructs the original mesh. Note that in certain cases, self-intersection between the edges must be introduced.

Supplementary Methods

A - Triangular mesh reconstruction

The regular pattern printed on the substrate is a perfect triangular mesh whose vertices are the QD nanodiscs (Fig. 1e). A custom built algorithm was used to define a mesh whose connectivity is identical to the original mesh (i.e. each vertex with exactly 6 neighbors). The algorithm proceeds in two steps, by firstly identifying regions with low distortion (where the original connectivity is easily recovered), and then filling the remaining regions through an advanced and computationally more expensive procedure:

Step 1

The low distortion regions are identified based on the properties of the as-printed mesh. Each QD nanodisc has exactly six neighbors at equal distance and forming angles of 60° between them. Each individual nanodisc is checked for this condition, and connected to its 6 neighbors only if the condition holds, up to a predefined tolerance to account for small deformations. Let p^i be the position of a nanodisc and p_1^i, p_2^i,…, p_6^i be its 6 closest neighbors (with respect to Euclidean distance) ordered counterclockwise around p^i. A nanodisc vertex is regular if and only if the standard deviation of all six angles and all six edge-lengths is smaller than 10 degrees and 250 nm respectively. Each detected regular vertex is now connected to its 6 neighbors, leading to a mesh that partially covers the image, as displayed in Supplementary Fig. 10a(left). This step robustly detects areas of the image featuring low distortions, while skipping the regions where the deformations are large (voids). At the end of this phase, all edges incident with non-regular vertices are removed.

Step 2

To fill the remaining regions (Supplementary Fig. 10a(right)), a procedure that finds the most uniform regular tessellation fitting the void, while snapping to the positions of nanodiscs, is used. Each void is a sequence of coordinates of vertices b_1,…, b_n at the boundary of the meshed region, in addition to a set of QD nanodisc positions d_1,…, d_m in its interior. For each boundary vertex b_i the number of regular vertices around it is known and denoted by n_i. The notation is illustrated in Supplementary Fig. 10b.

The void filling algorithm proceeds in 3 phases (Supplementary Fig. 10c): First, it identifies the connectivity of a triangulation that can fill the void (Supplementary Fig. 10c(II)), then warps the mesh to perfectly match the given boundary (Supplementary Fig. 10c(III)) and finally snaps its internal vertices to the nanodisc positions, while preserving the regularity of the mesh (Supplementary Fig. 10c(IV))

1- **Connectivity:** The connectivity of the mesh required to fill the void is uniquely identified by the number of neighbors of the vertices on the boundary of the void and can be extracted with a simple algorithm that walks over a regular triangulation. Since the number of regular vertices that need to be inserted to make the vertex regular is known, we can uniquely decide which part of the mesh needs to be copied. The boundary walk is described in Supplementary Fig. 10d: The first boundary edge connects to a boundary vertex with 4 connected neighbors. Thus a straight walk is necessary to find the next boundary vertex. The next vertex along the boundary has 5 neighbors implying a left turn. The next boundary

vertex has two connections, therefore a sharp right must be taken. The entire boundary can be walked following this procedure. At the end of the walk, a regular mesh with the same boundary geometry is cut out of the perfect grid.

2- **Boundary fitting**: Connectivity alone is not sufficient to close the void, since generally it overlaps with other triangulated regions of the original image. The regular mesh is thus deformed solving the following linear system:

$$\boldsymbol{Lx} = \mathbf{0} \; s.t. \, \boldsymbol{x}_i = \boldsymbol{b}_i \in 1 \dots n$$

where $\boldsymbol{x}$ is a vector containing the coordinates of the mesh vertices. The constraint fixes the boundary vertices to match the void boundary and $\boldsymbol{L}$ is the uniform Laplacian[1], defined as follows:

$$\boldsymbol{L}_{ij} = \begin{cases} -\sum_j \boldsymbol{L}_{ij} & i == j \\ 1 & i \; is \; a \; neighbor \; of \; j \\ 0 & otherwise \end{cases}$$

The boundary of the deformed mesh matches (due to the constraints) the boundary of the void, while each interior vertex is placed at the barycenter of its neighbors, thus keeping the shape as similar as possible to a regular grid, where this property trivially holds.

3- **QDs snapping:** After the boundary fitting, the interior vertices will not necessarily overlap with the internal nanodisc positions (Supplementary Fig. 10c(III)). The optimal assignment of vertices of the mesh to the position of nanodiscs is obtained through an algorithm that preserves their regular structure. This is done by explicitly encoding the assignment between the internal vertices of the mesh and the position of the internal nanodisc positions in a permutation matrix $\boldsymbol{P}$. The optimal permutation minimizes the following energy:

$$min_{\boldsymbol{P}} \; || \; \boldsymbol{LPD} \; ||_2$$

where $\boldsymbol{D}$ is a matrix containing the position of one of the internal nanodiscs in each row, and $\boldsymbol{P}$ is a permutation matrix (i.e. each row and column sum to 1, and each entry can be either 0 or 1). The energy can be rewritten to explicitly highlights the variables, which correspond to the entries of $\boldsymbol{P}$.

$$||\boldsymbol{LPD}||_2^2 = \sum_i ||\boldsymbol{LPD}_i||_2^2 = \sum_i \boldsymbol{D}_i \boldsymbol{P}^T \boldsymbol{L}^T \boldsymbol{LPD}_i = \widetilde{\boldsymbol{P}}^T \left(\sum_i (\boldsymbol{I} \otimes \boldsymbol{D}_i)^T \boldsymbol{L}^T \boldsymbol{L} (\boldsymbol{I} \otimes \boldsymbol{D}_i) \right) \widetilde{\boldsymbol{P}}$$

Where $\boldsymbol{M_i} \, \widetilde{\boldsymbol{P}} = \begin{bmatrix} (\boldsymbol{P}^T)_1 \\ (\boldsymbol{P}^T)_2 \\ \dots \end{bmatrix}$

This is a binary integer optimization in its canonical form and the globally optimal solution is found with the Gurobi solver[2]. The running time of the solver varies from a tenth of a second for small voids to a few minutes for large voids, but increases dramatically for regions containing more than 150 vertices. When such regions occasionally appear in our experiments the operator is asked to manually split them in two parts drawing a straight line (Supplementary Fig. 10e).

In all, this approach is a good approximation of the material behavior and it is very robust, as demonstrated in examples featuring a high deformation (Supplementary Fig. 10f). Each void is independently filled and if at the end of this procedure a void is multiply connected, the internal islands are removed to obtain simply connected regions.

B - Synthesis of quantum dots

Materials for quantum dot synthesis

Cadmium oxide (CdO, 99.999%, 48-0800) was purchased from Strem Chemicals (USA). n-dodecylphosphonic acid (DDPA, 98%, 08118) was purchased from Epsilon Chimie (France). 1-butanol (ACS Grade, 101990) was purchased from Merck KGaA (Germany). Diphenylphosphine (DPP, 98%, 252964), ethanol (96%, 02850), hexadecylamine (HDA, 90%, H7408), hexane (95%, 208752), methanol (99.9%, 34860), selenium shot (99.999%, 204307), sulfur (99.5%, 84683), trioctylphosphine (Technical TOP, 90%, 117854), trioctylphosphine (TOP, 97%, 718165), trioctylphosphine oxide (TOPO, 90%, 346187), 1-octadecene (ODE, 90%, O806), 1-octanethiol (98.5%, 471836), octylamine (99%, O5802), oleylamine (OAm, 70%, O7805), oleic acid (OLA, 90%, 364525), and zinc acetate (Zn(ac)2, 99.99%, 383317) were all purchased from Sigma Aldrich.

Preparation of core-shell precursors

Cadmium oleate stock preparation for core-shell synthesis: Cadmium oleate stock was prepared by combining 0.256 g of CdO, 2.6 mL of OLA and 20 mL ODE in a three-neck 100 mL round-bottom flask. The flask was degassed three times at room temperature to below 0.1 Torr at a stir rate of 800 rpm. After pumping, the mixture was returned to nitrogen and the temperature raised to 270°C to form a clear, colorless solution, followed by maintaining this temperature for 30 min. The temperature was then reduced to 150°C and 1.3 mL of degassed OAm were added to prevent solidification of the stock. The temperature was next reduced to 100°C, the mixture degassed for 30 min and transferred into a nitrogen glovebox for future use.

Zinc oleate stock preparation for core-shell synthesis: Zinc oleate stock was prepared by combining 1.1 g of Zn(ac)2, 3.8 mL of OLA and 21 mL ODE in a three-neck 100 mL round-bottom flask. The flask was degassed three times at room temperature to below 0.1 Torr at a stir rate of 800 rpm. After pumping, the mixture was returned to nitrogen and raised to 200°C to form a clear, colorless solution, followed by maintaining this temperature for 30 min. The temperature was next reduced to 150°C and 3.95 mL of degassed OAm were added to prevent solidification of the stock. The temperature was subsequently reduced to 100°C and the solution degassed for 30 min and transferred into a nitrogen glovebox for future use.

Preparation of core-shell green nanocrystals

Synthesis of core CdSe/ZnS nanocrystals with a composition gradient: The core-shell nanocrystals with a composition gradient were prepared by modifying a published procedure [3]. Briefly, 51.4 mg of CdO, 734 mg of Zn(ac)2 and 2.8 mL of OLA were combined in a three-neck 100 mL round-bottom flask. The mixture was degassed three times under 0.1 Torr, then raised to 150°C for 30 min with stirring at 1000 rpm. The flask was then switched to nitrogen, 20 mL of degassed ODE were added and the flask was pulled under vacuum at 120°C for an additional 10 min. The temperature was raised to 310°C to form a clear, colorless solution of cadmium oleate and zinc oleate. At 310°C, 3 mL of TOP (technical, 90%) containing 0.1 mmol selenium and 4 mmol sulfur were swiftly injected into the reaction mixture. The temperature was maintained at 300°C for 10 min, then cooled to room temperature. To purify the nanocrystals, the reaction mixture was transferred to a 50 mL centrifuge tube, filled with ethanol to yield a total volume of 50 mL, vortexed and centrifuged at 4000 rpm for 10 min. The centrifuge tube was left undisturbed until complete separation into a clear top phase

(byproducts) and colored bottom phase (nanocrystals). The top phase was removed with a syringe. The ethanol extraction procedure was repeated (typically five times) until a bright pellet was fully precipitated with a clear supernatant. The pellet was dispersed in 8 mL of hexane and stored in the dark until future use. The recipe resulted in core-shell nanocrystals with a first excitonic absorption at 516 nm and emission at 540 nm.

Growth of ZnS shell on CdSe/ZnS nanocrystals: The ZnS shell was grown on CdSe/ZnS nanocrystals following a modified published recipe [4]. Briefly, 340 µL of CdSe/ZnS nanocrystals in hexane at an optical density of 20 at the lowest energy excition, 3 mL of ODE and 3 mL of OAm were combined in a three-neck 100 mL round-bottom flask. The mixture was degassed at room temperature for 1 h and raised to 120°C for 20 min with stirring at 800 rpm. The mixture was switched to nitrogen and raised to 280°C at a rate of 16°C min^{-1}. At 210°C, two separate syringes of zinc oleate (0.25 mmol) and octanethiol (a 2-fold excess) precursors were each diluted in ODE to give a total volume of 3 mL and injected with a syringe pump at a rate of 2.5 mL $hour^{-1}$ for 72 min. After precursor injection, the temperature was lowered to 200°C and 1 mL of degassed OLA was added drop-wise. The mixture was next annealed for 1 h at 200°C. After annealing, the reaction flask was let to cool to room temperature. To precipitate and clean the nanocrystals, an equivalent amount of ethanol was added to the reaction mixture which was then centrifuged at 4000 rpm for 10 min. The clear supernatant was discarded. The bright precipitate was redispersed in 2 mL of hexane, and crashed out with 10 mL of ethanol at 4000 rpm for 10 min. The colorless supernatant was discarded. The precipitate was re-dispersed in 1 mL of hexane and crashed out with 10 mL of ethanol and 4000 rpm for 10 min. The clear (or slightly colored) supernatant was again discarded. The nanocrystals were dispersed in 3 mL of hexane and stored in the dark until future use. The recipe resulted in nanocrystals with a first excitonic absorption at 497 nm and an emission at 522 nm in hexane. Once mixed with Au nanoparticles and printed, the emission redshifts to 535 nm.

Preparation of core-shell blue nanocrystals

Synthesis of CdS/ZnS core/shell nanocrystals with a composition gradient: The core-shell nanocrystals with a composition gradient were prepared by modifying a published procedure [5]. Briefly, 128.4 mg of CdO, 1832 mg of Zn(ac)2 and 7 mL of OLA were combined in a three-neck 100 mL round-bottom flask. The mixture was degassed three times under 0.1 Torr, then at 150°C for 20 min while stirring at 1000 rpm. The flask was switched to nitrogen, 15 mL of degassed ODE was added and pulled under vacuum at 120°C for an additional 10 min. The temperature was raised to 305°C to form a clear, colorless solution of cadmium oleate and zinc oleate. At 305°C, 3 mL of ODE:S solution was swiftly injected into the reaction mixture. To prepare the ODE:S solution, 51.3 mg of sulfur was weighed in a glass vial, brought into a glovebox and 3 mL of degassed ODE was added. The mixture was heated on a hotplate at 225°C until all the sulfur dissolved (approximately 8 min) and the solution turned to a light yellow color. Right afterwards, the solution was cooled for 5 min and taken up in a syringe for injection.

The temperature of the reaction mixture was maintained at 310°C for 12 min after injection, then 5 mL of OLA with 4 mmol of sulfur was injected with a syringe pump at a rate of 0.5 mL min^{-1}. To prepare the OLA:S stock, 513 mg of sulfur was weighed in a glass vial, brought into a glovebox and 20 mL of degassed OLA was added. The mixture was heated at 225°C for 20 min to promote temporary dissolution of the sulfur in the OLA. The OLA:S was allowed to cool for 5 min, taken up in a syringe

and immediately pumped into the reaction mixture. After precursor injection, the temperature was maintained at 310°C for 3 h. After annealing, the reaction mixture was cooled to room temperature. To precipitate and clean the nanocrystals, the reaction mixture was split into two 50 mL centrifuge tubes. 20 mL of ethanol were added to each tube and centrifuged at 8000 rpm for 10 min. The clear supernatant was discarded. To redisperse the nanocrystals, 5 mL of hexane was added (10 mL total) and 1 mL of octylamine (2 mL total) was added and sonicated until redispersion. To crash out the nanocrystals, 20 mL of ethanol was added and centrifuged at 8000 rpm for 10 min. The clear supernatant was discarded. The cleaning process was repeated two more times with 5 mL of hexane (10 mL total) and 20 mL ethanol (40 mL total), with the supernatant becoming progressively more colored. The particles were dispersed in 5 mL of hexane and stored in the dark until future use. The recipe resulted in core-shell nanocrystals with emission at 457 nm in hexane. Once mixed with Au nanoparticles and printed, the emission redshifts to 458 nm.

Preparation of core-shell-shell red nanocrystals[6]

"Synthesis of CdSe core nanocrystals. CdSe cores with their lowest-energy absorption peak at 589 nm (approximately 4.1 nm diameter[7]) were synthesized by modifying a published procedure[8]. Briefly, 822 mg of CdO, 16.2 g of TOPO, 37 g of HDA, and 3.215 g of DDPA were combined in a 250-mL four-neck round-bottom flask and heated to 90 °C under N2. At 90 °C, the mixture was degassed three times to below 0.1 Torr (0.133 mbar) with stirring at 1000 r.p.m. The flask was returned to N2, heated to 320 °C until the solution turned clear and colorless, and held for 15 minutes. The temperature was reduced to 260 °C, the stir rate reduced to 300 r.p.m., and 8 mL of 1M Se in 97% TOP (1M TOP:Se) with 85 µL of DPP was swiftly injected. After injection, the stir rate was increased to 1000 r.p.m. and the temperature was maintained at 260 °C for 155 min to reach a size of 4.1 nm[7]. The reaction was quenched by removing the heating mantle, submerging the flask in a water bath when the reaction mixture reached 200 °C, and adding 40 mL of 1-butanol at 130 °C to prevent solidification.

The mixture was evenly split between six 50-mL centrifuge tubes, and methanol was then added to each tube to yield a total volume of 50 mL and centrifuged at 4000 r.p.m. for 10 min. The clear supernatant was discarded. The precipitate in each tube was redispersed in 20 mL of hexane (120 mL total) and left undisturbed overnight. The following day, the mixture was centrifuged once more at 4000 r.p.m. for 20 min to precipitate unreacted material. The supernatant was saved, transferred to six fresh 50-mL centrifuge tubes, precipitated with ethanol, and centrifuged at 4000 r.p.m. for 10 min once more. The supernatant was discarded. The precipitate in each tube was redispersed in 4 mL of hexane (24 mL total) and precipitated again with ethanol and centrifugation at 4000 r.p.m. for 10 min. The supernatant was discarded. To yield a concentrated dispersion, the nanocrystals were redispersed in 20 mL of hexane and stored in the dark until further use.

Growth of CdS/ZnS shell/shell on CdSe cores to obtain red-emitting QDs. The CdS/ZnS shell/shell was grown on CdSe cores following a published procedure[4]. Briefly, 100 nmol of CdSe cores in hexane, 3 mL of ODE, and 3 mL of OAm were added to a three-neck 100-mL round-bottom flask while stirring at 800 r.p.m. The mixture was degassed at room temperature for an hour, then at 120 °C for 20 min while stirring at 800 r.p.m. The mixture was switched to N2, then heated to 305 °C at a rate of 16 °C min^{-1}. At 210 °C, two separate syringes of cadmium oleate (0.22 mmol) and octanethiol (a 1.2-fold excess, 0.264 mmol) precursors were each diluted in ODE to give a total volume of 3 mL and injected with a syringe pump at a rate of 1.5 mL h^{-1} for two hours. After precursor injection, the temperature

was lowered to 200 °C, 1 mL of degassed OLA was added dropwise, and the mixture annealed for an hour at 200 °C.

After annealing, the temperature was lowered to 120 °C and the reaction mixture was degassed for 30 min under vacuum. After degassing, the flask was switched to N2 and the temperature was raised to 280 °C at a rate of 16 °C min^{-1}. At 210 °C, two separate syringes of zinc oleate (0.24 mmol) and octanethiol (a 2-fold excess) precursors were each diluted in ODE to give a total volume of 3 mL and injected with a syringe pump at a rate of 2.5 mL h^{-1} for 72 min. After precursor injection, the temperature was reduced to 100 °C, degassed for 10 min under vacuum, and then cooled to room temperature under N2.

To precipitate and clean the QDs, an equivalent amount of ethanol to reaction mixture was added and the combination was centrifuged at 4000 r.p.m. for 10 min. The clear supernatant was discarded, the bright precipitate redispersed in 4 mL of hexane, and precipitated again with 10 mL of ethanol and 4000 r.p.m. for 10 min. Again, the colorless supernatant was discarded, the precipitate redispersed in 2 mL of hexane, and precipitated with 5 mL of ethanol and 4000 r.p.m. for 10 min. The clear supernatant was discarded and the particles dispersed in hexane and stored in the dark until future use. The recipe resulted in core/shell/shell QDs with their lowest-energy absorption peak at 614 nm and an emission maximum at 627 nm in hexane."[6]

Supplementary References:

1. Sorkine O. Laplacian Mesh Processing. *STAR – State of The Art Report EUROGRAPHICS '05*, doi:10.2312/egst.20051044 (2005).

2. Gurobi Optimizer. Available from: www.gurobi.com

3. Bae WK, Char K, Hur H, Lee S. Single-step synthesis of quantum dots with chemical composition gradients. *Chem Mater* **20,** 531-539 (2008).

4. Boldt K, Kirkwood N, Beane GA, Mulvaney P. Synthesis of Highly Luminescent and Photo-Stable, Graded Shell CdSe/CdxZn1-xS Nanoparticles by In Situ Alloying. *Chem Mater* **25,** 4731-4738 (2013).

5. Lee KH, Lee JH, Song WS, Ko H, Lee C, Lee JH*, et al.* Highly Efficient, Color-Pure, Color-Stable Blue Quantum Dot Light-Emitting Devices. *Acs Nano* **7,** 7295-7302 (2013).

6. Kress SJ, Antolinez FV, Richner P, Jayanti SV, Kim DK, Prins F*, et al.* Wedge Waveguides and Resonators for Quantum Plasmonics. *Nano letters* **15,** 6267-6275 (2015).

7. Yu WW, Qu LH, Guo WZ, Peng XG. Experimental determination of the extinction coefficient of CdTe, CdSe, and CdS nanocrystals. *Chem Mater* **15,** 2854-2860 (2003).

8. Reiss P, Bleuse J, Pron A. Highly luminescent CdSe/ZnSe core/shell nanocrystals of low size dispersion. *Nano letters* **2,** 781-784 (2002).
